# A Survey on Salt Content Labeling of the Processed Food Available in Malaysia

**DOI:** 10.3390/ijerph17072469

**Published:** 2020-04-04

**Authors:** Hasnah Haron, Ivy Hiew, Suzana Shahar, Viola Michael, Rashidah Ambak

**Affiliations:** 1 Nutritonal Sciences Programme and Centre for Healthy Aging and Wellness, Faculty of Health Sciences, Universiti Kebangsaan Malaysia, Jalan Raja Muda Abdul Aziz, Kuala Lumpur 50300, Malaysia; ivyhiew29@gmail.com; 2 Dietetics Programme and Centre for Healthy Aging and Wellness, Faculty of Health Sciences, Universiti Kebangsaan Malaysia, Jalan Raja Muda Abdul Aziz, Kuala Lumpur 50300, Malaysia; suzana.shahar@ukm.edu.my (S.S.); rashidah662009@gmail.com (R.A.); 3 Non-communicable Disease Division, Ministry of Health, Putrajaya 62590, Malaysia; violamichael@moh.gov.my; 4 Institute of Public Health, Jalan Bangsar, Federal Hill, Wilayah Persekutuan Kuala Lumpur, Kuala Lumpur 50590, Malaysia

**Keywords:** salt, sodium, processed foods, labeling

## Abstract

Salt content in processed foods is high, and it is usually used as preservatives, stabilizers, and color enhancers in the products. Increased consumption of processed foods in the modern world has contributed to a high salt intake and thus increased the prevalence of hypertension among Malaysian populations. Therefore, this study aimed to identify and compare salt content in processed food products available in supermarkets and determine the percentage of processed food products exceeding the reference value stated in International Product Criteria (2016). The percentage of processed food products without salt and sodium labeling was determined in this study, in which 76.5% of unlabeled processed food products were made in Malaysia, while 23.5% were imported products. The food group with the highest average salt content was gravy and sauce (3.97 g/100 g), followed by soup (2.95 g/100 g), cheese (2.14 g/100 g), meat (1.37 g/100 g), fish (1.25 g/100 g), chicken (1.20 g/100 g), vegetables (1.18 g/100 g), butter and margarine (1.13 g/100 g), breakfast cereal (0.94 g/100 g), savory snacks (0.90 g/100 g), flatbread (0.86 g/100 g), sweet snacks (0.30 g/100 g), and potato (0.29 g/100 g). In addition, 79.5% of butter and margarine products had an average salt content above the reference value stated in the International Product Criteria, followed by gravy and sauce (79.3%), vegetables (72%), soup (50%), fish (49.2%), breakfast cereal (41%), cheese (36.6%), potato (36%), savory and sweet snacks (29.1), meat (12.5%) and chicken products (2.3%). Most processed food products available in local supermarkets were high in salt content.

## 1. Introduction

Salt is an inorganic compound consisting of sodium ions and chloride ions [1]. It is used to prevent the growth of bacteria and microorganisms as well as a natural preservative to prolong the shelf-life of food products in the market [2]. Therefore, sodium appears in high content among processed foods [3,4]. Malaysians are exposed to high-salt diet where too much salt has traditionally been added during cooking, which was initially added in as a flavor. Apart from that, Malaysians’ high-salt diet also due to high consumption of processed food. According to Sharif et al., salt was found as the main additive in every sample of processed food used in their study [5]. The processed food groups containing the highest amount of sodium were sauces and spreads (1090 mg/100 g), snacks (739 mg/100 g), and processed meats (590 mg/100 g) [6].

High sodium intake is associated with uncontrolled hypertension [7]. According to the Malaysian Health Minister Dr. Subramaniam, excessive salt intake causes hypertension, which in turn leads to heart diseases, stroke, and eventual death [8]. Furthermore, there is scientific evidence linking excessive sodium intake to cardiovascular disease, stomach cancer, osteoporosis, cataracts, and kidney stones [9]. Prevalence of hypertension among rural Malays in Malaysia was 30.3% [10]. Further, according to the National Health and Morbidity Survey, the prevalence of hypertension among adults aged 18 and above was ~30% [11]. Therefore, WHO has advised adults to consume no more than one teaspoon of salt per day, equivalent to 5 g of salt or 2000 mg of sodium [12]. However, a study conducted among Ministry of Health (MOH) staff showed salt intake of 7.15 g per day, exceeding the WHO recommendation [13]. In addition, another study conducted in Malaysia involving young adults at university also reported salt intake that exceeds WHO recommendations, which was 10.8 g per day [14].

In Malaysia, mandatory labeling of sodium and salt in processed food products is not required [15]. Although the Ministry of Health in Malaysia has been expediting mandatory labeling of sodium content on food products, this regulation has not been launched to date [8]. Community exposure to a high-salt diet has contributed to a high salt intake among Malaysian population which in turn has led to a high prevalence of hypertension in Malaysia. This worrying condition in Malaysia should be taken seriously. In addition, sodium content of food found in Malaysian food composition (MyFCD) is limited. There were a lot of processed food products found with no sodium content in MyFCD, such as chicken nuggets and tuna chili [16]. Thus, it is important to identify and compare salt content among processed food products available in the market to determine the percentage of processed food products exceeding the reference value stated in International Product Criteria (2016). Effort has been made to determine sodium content in soy sauces available at top supermarkets in the capital city of Malaysia [17]; however, that study did not include other types of processed foods. Thus, this present study aimed to identify salt content on the label of processed foods (excluding soy sauces) in Kuala Lumpur, the capital city of Malaysia.

## 2. Materials and Methods

### 2.1. Study Design

This study was a market survey on the sodium and salt content in nutrition labels for processed food products available in the main supermarkets located at Kuala Lumpur. A total of 938 processed food products were included in this study.

### 2.2. Sampling of Processed Food Products

Processed food products are defined as food products other than raw agricultural commodities that have been washed, cut, heated, pasteurized, and cooked, involving canning, freezing, drying, mixing, packaging, or other procedures that alter the food from its natural conditions [18].

### 2.3. Research Instruments

All data were collected using the nutrition labels found in the processed food products.

### 2.4. Nutrition Labels

The survey of sodium labeling in processed food products was conducted in Giant, Tesco, JayaGrocer, Mydin, and Econsave supermarkets. These five supermarkets were selected due to frequent visits by consumers of different socioeconomic statuses. Therefore, the processed food products involved in this study included all the processed food products commonly consumed by consumers of all socioeconomic statuses. A picture of the nutrition information panel on the package was taken using a camera handphone. The collection of information was carried out based on the name, brand of the products, amount of sodium per serving in milligrams (mg), amount of salt per serving in grams (g), amount of sodium per 100 g of sample (mg), and amount of salt per 100 g sample (g).

### 2.5. Statistical Analysis

Statistical Package for Social Sciences (SPSS) version 23.0 was used to analyze the data. Descriptive tests were used to identify the mean and standard deviation of salt content for each processed food group. Descriptive tests were also used to identify the number and percentage of processed food products exceeding the sodium criteria stated in International Product Criteria (2016) as well as the percentage of processed food products without sodium and salt labeling.

## 3. Results

A total of 938 products were included in this study, consisting of 142 processed chicken products, 195 processed fish products, 30 processed meat products, 58 vegetable products, 62 breakfast cereals, 30 potato-based products, 30 soup products, 116 gravies and sauces, 113 cheeses, 44 butter and margarine products, 76 savory snacks (pie, lasagna, pizza, popiah, curry, samosa, pau, and dumpling), 20 sweet snacks (pau and glutinous wheat), and 22 flatbreads (paratha, chapatti and roti canai). However, only 632 products had sodium or salt labeling, and the remaining 306 products (32.6%) had no labeling of sodium and salt. Table 1 shows the number of processed food products with and without sodium and salt labeling. 

Table 2 shows the number and percentage of processed food products with and without labeling of sodium and salts. Based on this table, there were 528 products from Malaysia that were collected in this study, with 294 (55.7%) products having sodium or salt labeling and 234 (44.3%) products without sodium and salt labeling. The number of imported products with sodium or salt labeling was higher (338 products, 82.4%) than the number of imported products that were without the labeling (72 products, 17.6%). Overall, there were 32.6% (306 products) of processed food products available in the supermarket that did not have sodium and salt labeling. Out of these 306 processed food products, 76.5% of them were local products and 23.5% were imported. 

Table 3 shows the average sodium and salt content per 100 g in the processed food groups. Results showed that gravy and sauce was the food group with the highest amount of salt content (3.97 g/100 g), followed by soup (2.95 g/100 g), cheese (2.14 g/100 g), meat (1.37 g/100 g), fish (1.25 g/100 g), chicken (1.20 g/100 g), vegetables (1.18 g/100 g), butter and margarine (1.13 g/100 g), breakfast cereal (0.94 g/100 g), savory snacks (0.90 g/100 g), flatbread (0.86 g/100 g), sweet snacks (0.30 g/100 g), and potato (0.29 g/100 g).

There were 632 products that exceeded the sodium criteria stated in International Product Criteria (2016) as shown in Table 4. Results showed that 79.5% of butter and margarine products exceeded the sodium criteria, followed by 79.3% of gravies and sauces, 72% of vegetables, 50% of soup products, 49.2% of fish products, 41% of breakfast cereals, 36.6% of cheese products, 36% of potato products, 29.1% of sweet and savory snacks, 12.5% of meat products, and 2.3% of chicken products. 

## 4. Discussion

Based on Table 1, most fish products, gravies, sauces, potatoes, breakfast cereals, butters, margarines, cheeses, savory snacks, sweet snacks, and flatbreads had labeling of sodium or salt. However, for the chicken products, only 44 products (31%) contained sodium or salt labeling. For meat products, only eight products contained sodium or salt labeling. For the soup products, there were 18 products with labeling of sodium or salt, and 12 products were without labeling. For vegetable products, 33 products were found without sodium and salt labeling.

Table 2 shows the number of imported products with sodium or salt labeling was higher than the number of imported products that were without labeling. This was likely due to the non mandatory regulation for labeling of sodium and salt content in Malaysia, which is mandatory in other countries such as the United Kingdom [19,20]. Therefore, most imported products contained sodium or salt labeling, but a limited number of Malaysian food products had this labeling. Salt labeling in food products can benefit consumers, as they can choose products with lower salt content with the help of the nutrition labeling [21]. Systematic surveys in the United Kingdom also reported that if the labeling system was clear and easy to understand, strategies for reducing salt intake might be effective by implementing nutrition labels [22]. Therefore, labeling of sodium or salt is one of the most effective strategies in reducing sodium intake among Malaysian populations which exceeded the WHO recommendation level [13,14,23].

Salt is used to prevent the growth of bacteria and microorganisms and act as a natural preservative that prolongs the shelf-life of food products in the market [2]. Therefore, processed food products contained a higher amount of salt or sodium. Table 3 shows the average sodium and salt content per 100 g in the processed food groups like gravy and sauce was the food group with the highest amount of salt content. This result was supported by the results of several previous studies which reported that sauce and processed meat were the food groups with the highest level of salt content [3,6]. According to Charlton et al. dairy products contributed to 11% of sodium intake in the diet [24]. In accordance, cheese was the dairy product with the third highest salt content according to the data from this study. According to Doyle and Glass, sodium reduces water activity in food and thus limits the pathogen growth [25]. For example, sodium chloride limited the growth and release of toxins by *Clostridium botulinum* in cheese [26]. Sodium reduction in cheese could affect starter culture activities [27]. Therefore, sodium content in cheese products is high. In addition to daily, processed meat is also a main source of salt intake [28]. Sodium is added to processed meat to bind and maintain the water content in these meat products [29]. Sodium reduction might affect the texture and other qualities of the meat products, such as moisture levels, fat content, and pH level [30]. Therefore, sodium content in processed meat products is also high.

According to the Health Minister of Malaysia, Datuk Seri Dr. S. Subramaniam, in the year 2017, the Ministry of Health Malaysia worked with the food industry to reformulate food products for the purpose of lowering salt content in processed food, including for gravy and soup products as well as frozen meat products with the highest salt content. However, according to Lucas et al., significant sodium reduction of more than 50% could affect consumer acceptance on food [31]. In addition, sodium reduction might increase the risk of undesirable bacterial growth and shorten food shelf-life [25]. When sodium is reduced, other preservatives might be used for all newly developed sodium-free products to ensure microbiological safety and storage life [31]. Reduction in sodium had to be combined with the use of other additives so that consumer acceptance remains unaffected and the risk of bacterial growth is lowered [31].

The World Health Organization (WHO) had taken action by publishing a Global Action Plan proposing a reduction of 30% salt intake by 2025 [32]. However, previous studies showed that sodium intake among Malaysian populations exceeded the WHO recommendation level [13,14,23]. Processed foods contributed substantially to the salt intake in a person’s diet [33]. This indicated that even though the population was trying to reduce their salt intake by not adding salt, they would still consume a substantial amount of salt through processed foods. Therefore, the most effective way of reducing salt intake was to reduce the amount of salt in processed foods [12,33]. Thus, International Product Criteria (2016) serves as a guide to reduce the amount of salt in processed food products. There were 632 products in this study that exceeded the sodium criteria stated in International Product Criteria (2016) as shown in Table 4. According to Bertram et al., the involvement of companies that produce processed food products is required to implement a comprehensive salt reduction plan [34].

Previously, no study has surveyed salt content in processed foods in processed, canned products and frozen food products in Malaysia. However, we have included a large sample size and actual processed food which consisted of 938 products. The main weakness of our study would be the reliance on salt data from package labels reported by the manufacturer, which is assumed to be accurate. Therefore, for further studies, laboratory tests can be conducted to identify the salt content of processed food products available without the labeling of sodium and salt.

## 5. Conclusions

In conclusion, 76.5% of unlabeled processed food products were made in Malaysia, while 23.5% were imported products. Among the 13 food groups involved in this study, gravy and sauce had the highest salt content, followed by soup, cheese, and meat products. Based on the data of this study, more than 70% of available butter, margarine, gravy, sauces, and vegetable products exceeded the sodium criteria stated in International Product Criteria 2016.

## Figures and Tables

**Table 1:** Number and percentage of processed food with and without labeling of sodium and salt.

Processed Food Groups	Sodium/Salt Labeling			
	Yes		No	
	Number (n)	Percentage (%)	Number (n)	Percentage (%)
Chicken (n = 142)	44	31	98	69
Fish (n = 195)	122	62.6	73	37.4
Meat (n = 30)	8	26.7	22	73.3
Vegetable (n = 58)	25	43.1	33	56.9
Potato (n = 30)	25	83.3	5	16.7
Breakfast cereal (n = 62)	61	98.4	1	1.6
Soup (n = 30)	18	60	12	40
Gravy and sauce (n = 116)	92	79.3	24	20.7
Butter and margarine (n = 44)	39	88.6	5	11.4
Cheese (n = 113)	112	99.1	1	0.9
Savory snacks (n = 76)	52	68.4	24	31.6
Sweet snacks (n = 20)	19	95	1	5
Flat bread (n = 22)	15	68.2	7	31.8

**Table 2:** Number and percentage of processed food products manufactured in Malaysia and imported with and without labeling of sodium or salt.

Sodium/Salt Labeling	Malaysia Products		Imported Products		Total	
	Number	Percentage (%)	Number	Percentage (%)	Number	Percentage (%)
**Yes**	294	55.7	338	82.4	632	67.4
**No**	234	44.3	72	17.6	306	32.6
**Total**	528	100.0	410	100.0	938	100.0

**Table 3:** The average sodium and salt content in processed food groups reported in mean ± standard error (SE)

Processed Food Group	Sodium Content (mg/100 g) (mean ± SE)	Salt Content (g/100 g) (mean ± SE)
Gravy and sauce (n = 92)	1589.75 ± 1544.67	3.97 ± 3.86
Soup (n = 18)	1273.33 ± 2003.18	2.95 ± 4.75
Cheese (n = 112)	856.54 ± 374.74	2.14 ± 0.94
Meat (n = 8)	546.50 ± 260.04	1.37 ± 0.65
Fish (n = 122)	499.85 ± 239.15	1.25 ± 0.60
Chicken (n = 44)	479.42 ± 186.33	1.20 ± 0.47
Vegetable (n = 25)	470.44 ± 543.34	1.18 ± 1.36
Butter and margarine (n = 39)	453.36 ± 276.13	1.13 ± 0.69
Breakfast cereal (n = 61)	377.33 ± 195.75	0.94 ± 0.49
Savory snacks (n = 52)	361.76 ± 159.74	0.90 ± 0.40
Flat bread (n = 15)	345.17 ± 127.56	0.86 ± 0.32
Sweet snacks (n = 19)	118.23 ± 117.83	0.30 ± 0.29
Potato (n = 25)	115.08 ± 136.75	0.29 ± 0.34

**Table 4:** Percentage of processed food products exceeding the sodium content in International Product Criteria (2016).

Processed Food Groups	Criteria of Sodium (mg/100 g) (International Product Criteria 2016)	Food Products Exceeds the Sodium Content in International Product Criteria (n)	Percentage of Food Products Exceeding Sodium Content in International Product Criteria (%)
Butter and margarine (n = 39)	≤160	31	79.5
Gravy and sauce (n = 92)	≤450	73	79.3
Vegetables (n = 25)	≤100	18	72.0
Soup (n = 18)	≤300	9	50.0
Fish (n = 122)	≤450	60	49.2
Breakfast cereals (n = 61)	≤400	25	41.0
Cheese (n = 112)	≤830	41	36.6
Potatoes (n = 25)	≤100	9	36.0
Snacks (sweet and savory) (n = 86)	≤400	25	29.1
Meat (n = 8)	≤820	1	12.5
Poultry (n = 44)	≤820	1	2.3
